# Parental and Dentist Satisfaction with Primary Anterior Zirconia Crowns: A Case Series Analysis

**DOI:** 10.3390/children8060451

**Published:** 2021-05-26

**Authors:** Lawrence Yanover, William Waggoner, Ari Kupietzky, Moti Moskovitz, Nili Tickotsky

**Affiliations:** 1 Private Practice, 126 Lakeshore Rd, St. Catharines, ON L2N2T5, Canada; 2 Private Practice, 8981 West Sahara Ave #110, Las Vegas, NV 89031, USA; wwaggoner1@gmail.com; 3 Department of Pediatric Dentistry, Hebrew University Hadassah School of Dental Medicine, P.O. Box 12272, Jerusalem 9112102, Israel; drkup@netvision.net.il (A.K.); motim@md.huji.ac.il (M.M.); 4 Department of Immunology, Weizmann Institute of Science, Rehovot 76100, Israel; nilitiko@gmail.com

**Keywords:** pediatric dentistry, restorative dentistry, zirconia pediatric crowns

## Abstract

This retrospective cohort study evaluated overall parental satisfaction of zirconia crowns (ZC) placed on primary maxillary anterior teeth with that of two independent, blinded dentists. 131 ZC placed in 37 children, aged 24.8–62.2 months (mean = 42.8), who had at least one recall visit a minimum of 6 months after placement were rated (average = 13.3). Crown colour match, crown contour and crown durability were evaluated by parents and compared to photographic evaluations of two independent raters. Overall parental satisfaction was also evaluated. The overall retention rate was 99.7% and parental satisfaction was 100%. Colour match was rated excellent by 84% of parents and 36% of dental evaluators. Crown contour was rated excellent by 97% of parents and 55% of dental evaluators. The length of follow-up had no effect on colour match or crown contour. ZC comprises an aesthetic and durable option for restoring carious primary maxillary incisors and were well-accepted by parents. Parents were less critical than dental evaluators of crown appearance.

## 1. Introduction

There is increasing demand from parents for dentists to provide improved aesthetic solutions when restoring their children’s teeth and there are an increasing number of options to provide this treatment [1,2]. Where minimal caries is detected, discing of affected interproximal caries may be carried out along with improved oral hygiene, topical fluoride application and reduced snacking on fermentable carbohydrates. A minimal invasive dentistry approach might include the use of silver diamine fluoride to arrest decay but this leaves carious lesions blackened and lacks aesthetic appeal. Repair of small carious lesions can be approached with cosmetic materials such as resin-modified glass ionomers or bonded composite resins. Aesthetic full crown coverage for primary anterior teeth for teeth severely affected by early childhood caries or with a high risk of recurrent caries, would include full coverage restorations bonded onto the tooth such as resin composite strip crowns (RCSC) and crowns that are retained on the tooth by cementation. The latter include pre-veneered stainless steel crowns (VSSC) and the newer zirconia crowns [1,3,4]. The RSCS has excellent aesthetics, multiple resin shades, the ability to fit in crowded spaces and ease of repair [5,6]. The VSSC is less technique-sensitive but has a labial veneer with fewer shades, a facing that can debond and no ability to adjust crown contour [7,8].

Preformed anterior zirconia crowns (ZC) were introduced in 2008 to offer another aesthetic option for the restoration of primary anterior teeth. Zirconia crowns are colour-stable, resistant to fracture or debonding [9,10], biocompatible [11,12] and autoclavable if contaminated [13] but come in limited shades and shapes. Sizes must be carefully selected to fit the given arch space and existing tooth size and shape because they cannot be easily altered. They only come in one or two shades so this must be carefully evaluated for ideal esthetic results. The tooth must be reduced to accept the crown size selected as zirconia cannot be adjusted to provide an acceptable fit. With proper technique, haemorrhage and moisture contamination can be controlled prior to luting, as cementation will be affected by a contaminated field similar to what is expected for the bonding of resin materials. The crowns must be held in alignment while the cement cures, otherwise esthetics are impacted thus patient compliance is required, although this is simpler and faster than that required for RSCS [1,4].

Acceptance of ZC continues to grow in the restoration of decayed primary anterior teeth, which is due to a number of factors. The cost of providing treatment is similar to alternate full coverage options dependent upon local factors. Durability is better than popular RSCS, including fracture resistance, marginal integrity and colour stability [14,15]. With increasing clinician acceptance of ZC, it is important to evaluate the parental satisfaction of restorative options. In previous studies, the use of ZC for restoration of primary anterior teeth has met parent approval and was more widely accepted than VSSC and RSCS [9,15,16]. The main aim of this retrospective study was to evaluate parental satisfaction with the clinical appearance of ZC placed on their children placed by a single provider. Our secondary aim was to compare parental satisfaction with the ratings of independent dental examiners. We also hoped to evaluate if the three crown brands used influenced parental satisfaction.

## 2. Materials and Methods

The convenient sample comprised of 131 zirconia crowns placed in 37 children who had carious primary incisors with extensive caries (evaluated by clinical and radiographic examination) on a single surface or moderate carious lesions on two or more surfaces. The study group comprised 27 boys and 10 girls, aged 24.8–62.2 months (median = 40.6 months). A single practitioner (LY) experienced with placing ZC completed all of the restorations and conducted all clinical evaluations between 2015 and 2018.

Inclusion criteria: Included in the study were all children who had zirconia crowns placed by the same practitioner between January 2015 and March 2018 and had at least one recall visit at least 6 months after placement.

The study protocol was approved by the Institutional Human Subjects Ethics Committee, Hebrew University, Hadassah School of Dental Medicine. All procedures performed were in accordance with the ethical standards of the institutional and national research committee (Reference number 0554-18-HMO). Informed consent was obtained from all parents/legal guardians of participating subjects to allow their information to be used in the study.

### 2.1. Treatment Procedure

Patient behaviour management utilized oral sedation with hydroxyzine and nitrous oxide inhalation to treat 16 patients (78 crowns) and general anaesthesia was selected for 21 patients (53 crowns). Local anaesthesia was administered for all patients (lidocaine 2% with 1:100,000 epinephrine, Dentsply Sirona, Canada) followed by rubber dam placement and crown preparation. Teeth were prepared following manufacturer recommendations, using a standard protocol incorporating a retentive design that included parallelism, retentive areas left by caries removal and the use of horizontal striations on smooth surfaces to enhance retention. If caries had reached the pulp, a formocresol pulpotomy was performed [17], followed by zinc oxide eugenol filling over the pulp stumps.

More than one kind of zirconia crown was utilized. Crown brand selection was dictated by the facility location where the crowns were placed and included EZCrowns (Sprig Oral Health Technologies; Loomis, CA, USA), NuSmile (NuSmile; Houston TX, USA) and Cheng crowns (Cheng Crowns; Exton, PA, USA). When multiple crowns were placed, the same brand was used for all restorations. With NuSmile crowns, pink try-in crowns were used to confirm fit and white uncontaminated crowns were used for cementation. With other manufacturers, the actual crown was used for a trial fit and once selected, cleaned with Ivoclean (Ivoclar Vivadent; Mississauga, ON, Canada) prior to cementation to remove salivary and hemorrhagic contaminants and maintain bond strength to zirconia [18]. After tooth preparation and size selection, gingival bleeding was controlled by delaying cementation until another treatment was completed as well as applying pressure. The teeth were then rinsed and dried, followed by crown cementation according to manufacturer guidelines. Fuji Plus cement (GC America; Alsip, IL, USA) was placed in 124 crowns while Link Ace (GC America; Alsip, IL, USA) was placed in 5 teeth and BioCem (NuSmile; Houston, TX, USA) was placed in 2 crowns. Teeth were photographed preoperatively and crowns were photographed immediately postoperatively using an Olympus TG 4 camera on macro setting and no flash (Olympus America; Bethleham, PA, USA). When the child was due for a recall examination, postoperative radiographs were obtained and a clinical examination that included photographing the restored maxillary anterior teeth was completed.

To assess parental satisfaction, a survey of the parents of participating subjects was conducted at the same recall examination the photographs were obtained. Parents were given a questionnaire to complete at chair-side with the patient present, and asked to score parameters such as crown color, size, durability and their overall satisfaction with the crowns (Table 1), using a rating similar to the clinical photographic assessment done by the dentists [19]. Parents had the ability to look clearly at their child’s restored dentition at this time. They were also asked if they would choose this procedure again for their child or recommend it to a friend. The dentist who provided treatment (LY) was also present chair side to clarify any issues raised during the completion of the questionnaire.

The photograph rating system used by the two independent dental evaluators has previously been published in two evaluation studies of RCSC [20,21] and one study of ZC [19]. Each dentist received the digital clinical photographs and rated the clinical result independently. The results of each dentist evaluation were reviewed and where there was any disagreement, the consensus was reached through discussion.

**Figure 1:**
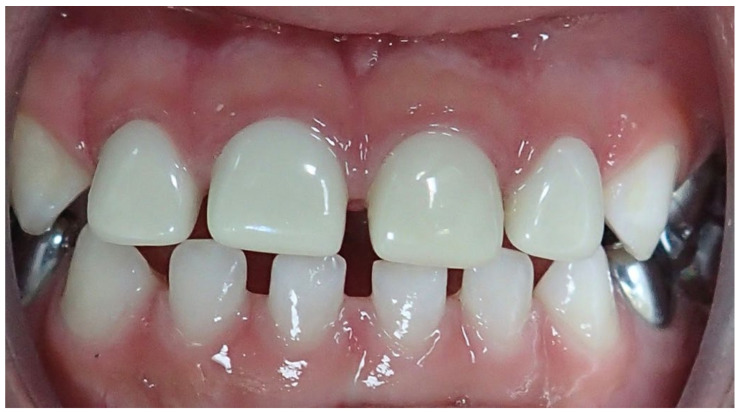
Restoration of four anterior teeth with pulp therapy on one tooth. Parent rated teeth 52, 51, 61, 62 as “slight mismatch from adjacent teeth”. Crown contour was “nicely contoured and natural looking”. Dentists rated teeth 52, 51 as “slight shade mismatch” and 61, 62 as “no noticeable difference from adjacent teeth”. Tooth 51 had a pulpotomy. Dentists rated 52 crown contour as “acceptable but could have been contoured better, perhaps longer, shorter, wider, thinner. Teeth 51, 61, 62 “appear very cosmetic, nicely contoured and natural looking.

**Figure 2:**
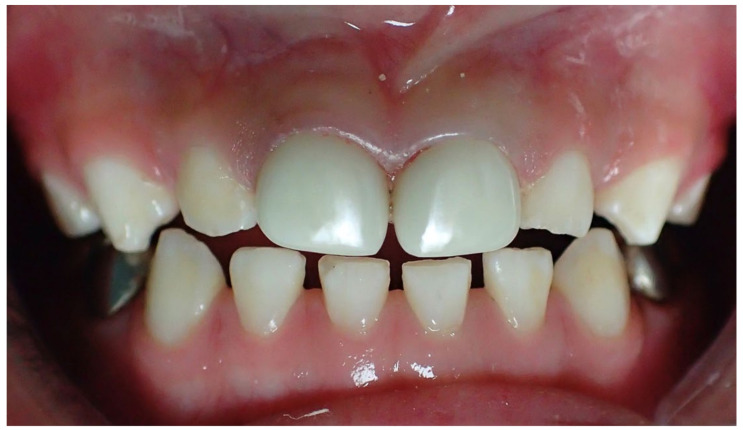
Restoration of two anterior teeth. Parents rated tooth 51 and 61 colour match as “no noticeable difference from other teeth”. Crown contour was “nicely contoured and natural looking”. Dentists rated colour match as “slight shade match” and crown contour as “not aesthetic, detracts from the appearance of mouth”.

The photographic assessment categories (Table 1) in this study were as follows: colour match was rated 1 for “no noticeable difference from adjacent teeth”, 2 for a “slight shade mismatch” and 3 for an “obvious shade mismatch”. Crown contour was rated 1 appearing “very cosmetic, nicely contoured and natural-looking”, 2 for “acceptable appearance but could have been contoured better, perhaps longer, shorter, wider, thinner”, 3 for “not aesthetic, detracting from the appearance of the mouth”, 4 for “not present”. Crown durability was rated 1 if the “crown appears normal; no cracks, chips or fractures”, 2 for “small but noticeable areas of loss of material”, 3 for “large loss of crown material” and 4 for “complete loss of crown”. The parental assessment was modified slightly for simplicity. Colour-match rating was identical for the parent survey. Crown contour was simplified to rating 1 for “nicely contoured and natural-looking”, 2 was “acceptable but could have better shape” and 3 as “unacceptable”. Examples of clinical cases are seen in Figure 1 and Figure 2. Crown durability was simplified to rating 1 for “crown appears intact with no chips, cracks or fracture”, 2 for “small, noticeable chips, cracks or fracture”, 3 for “large chips, cracks or fracture” and 4 if “crown is missing”. When a crown and tooth were missing, the response was 1 for “tooth lost naturally”, 2 for “trauma” and three was “extraction due to infection”. Overall satisfaction was evaluated as follows with a yes or no response to the questions; “Overall, were you satisfied with the results of the crowns?”. “Would you choose this procedure if once again offered?”. “Would you recommend this procedure to a friend with a child having a similar problem?”. The results of the parent questionnaire and that of the independent dental evaluators (AK and WW) were then compared.

### 2.2. Statistical Analysis

For the dentist evaluators and parental assessment groups, analysis was performed comparing the two groups, followed by comparison within each group by stratifying into two intervals: 22 patients followed for a short term interval of less than twelve months (6–10.4 months, mean 8.1 months) and 15 patients followed for a long term interval of at least 12 months (12.5–33.8 months, mean 21.0 months). Chi-square statistical analysis was used to compare the dentist evaluators and parent assessments of colour match and crown contour. This was also done within each group to determine the effect of time from treatment on the assessment values of crown colour match and contour.

## 3. Results

One hundred and third-one crowns with a follow-up ranging from 6 to 33.8 months (mean of 13.5 months) were evaluated (Table 2). Twenty- eight children had all four incisors restored, one child had three teeth restored as one was extracted and eight children had only two central incisors restored. Fifteen teeth of eight children had pulp therapy performed. The results include the evaluation of aesthetics and durability of ZC at 6–33.8 months follow-up visits from photographic assessment as well as from the parent questionnaire, completed by all 37 parents. Colour match (Table 3) was rated by 84% of parents as “no difference from the natural teeth” and 26% as “a slight mismatch” while the dental evaluators deemed 36% as “no noticeable difference from natural teeth”, 60% as “a slight mismatch” and 4% as “an obvious mismatch”. The parents more frequently ranked the crowns as “no difference from the natural teeth” and this difference was statistically significant (Chi-square *p* < 0.05). Crown contour (Table 4) was rated 97% “nicely contoured and natural-looking” by parents and 3% as “acceptable” while the dental evaluators rated 55% “very cosmetic, nicely contoured and natural-looking”, 36% as “acceptable but could have been contoured better” and 9% as “not aesthetic”. The parents more frequently ranked the crowns as “nicely contoured and natural-looking” and this difference was statistically significant (Chi-square, *p* < 0.05). Secondary results from the parental assessment and dentist evaluated photographs (Table 5) showed assessment values of the crown colour match were not associated with length of follow-up time (Chi-square, *p* < 0.05). In other words, the colour match appeared stable regardless of the length of time before evaluation. Regarding crown durability, neither parents nor dental evaluators observed any cracks, chips, fractures or loss of material. Only one crown debonded during the study and this was at two months after placement, due to a cement-to-tooth failure. An identical crown was recemented with no further issues. This tooth was included in the results.

Within the group of 8 patients with 15 of 28 teeth having pulpotomy, parents rated non-pulpotomized tooth colour at 35.7% having “no difference from natural teeth” and 10.7% having “a slight mismatch” while pulpotomized tooth colour was rated 35.7% having “no difference from natural teeth” and 17.9% having “a slight mismatch” (Table 6). Dental evaluators rated non pulpotomized tooth colour at 17.9% having “no difference from natural teeth” and 28.6% having “a slight mismatch”. Pulpotomized tooth colour was rated as 7.1% “no difference from natural teeth” and 46.4% as having “a slight or obvious mismatch” (Table 6). The “obvious mismatch” was only in one subject who had two central incisors treated. Even though there was a trend of pulpotomized teeth having slightly more colour mismatch, there was no statistically significant difference in colour match between pulpotomized and non-pulpotomized teeth noted by either parents or dental evaluators (Chi-square, *p* < 0.05).

All but one parent completed the overall satisfaction part of the questionnaire. All respondents were satisfied with the result of the crowns. They would choose this procedure if once again offered and would recommend this procedure to a friend with a child having a similar problem.

## 4. Discussion

Parents were more likely to rate both colour match to adjacent teeth and crown contour significantly better than the dental evaluators, who evaluated from digital photographs. Positive parental judgement of their child’s restored teeth may take into account the original appearance of these teeth which are often disfigured and blackened and thus the parents feel satisfied with the improvement they witness. The dentist examiner judges the result according to a detailed list of expected outcomes and is not emotionally involved in acceptance of any outcome. The photographic evaluation also allowed a longer and more detailed evaluation of the clinical result. This may have led to the dental evaluators being more critical of colour match than the parents. Parents may have been favourably influenced by the low failure rate of the crowns, with only one crown lost, with a simple resolution by cementing a new crown [16]. The crown failure was due to cement-to-tooth failure which can be reduced by controlling tooth surface contamination, careful case selection and conservative tooth reduction [4,19]. 

The effect of a short (6 to 10.4 month) or long (12 to 31.6 month) time period between treatment and examination by either parents or dentist for colour match did not detect any significant deterioration in colour match with time. The number of crowns rated for colour match as either “no difference from natural teeth” or “slight or obvious mismatch” was not significantly different between patients assessed fairly soon after placement or much longer after placement. Because the length of time the crowns were in the mouth did not affect the perception of colour match by parents or dental evaluators, this would seem to indicate there is very good colour stability of ZC, which has been observed elsewhere [15]. It should be noted that the mean-time before examination between the groups was not deemed significantly different as the number of subjects in each group was quite small.

When the effect of pulpotomy on tooth colour match was evaluated, there was no statistical difference noted between pulpotomized and non-pulpotomized treated teeth by either the parents or dental evaluators. Only one obvious shade mismatch was noted by the dental evaluators for a case where only two central incisors were treated with a pulpotomy. Pulpotomy has been shown to affect crown colour match in a study of RCSC [21]. Two other studies evaluating anterior ZC did not include teeth that had received pulpotomy so they did not report on the issue [15,16]. It would be prudent to leave pulpal medicaments below the gingival margin and place tooth coloured glass ionomer or similar material in the supragingival tooth restoration to best match the existing tooth to minimize any possible effect of pulpotomy on the final crown colour match. Anecdotally, ZC have been placed over teeth treated with silver diamine fluoride (SDF), which results in black areas of arrested decay without apparent shade problems, but the effect of SDF on ZC shade might be worth further investigation.

This study was limited by a small sample size of 37 patients and 131 teeth as well as an average follow-up period of 13.5 months. The difficulty of retrospective studies in a population of lower-income families with young children includes difficulty in getting timely and consistent follow-up for recall examinations, no interest in further dental care or a return to the family dentist for routine preventive visits. Furthermore, clinical observation of primary anterior teeth provides a fairly short window between treatment and exfoliation, limiting study time [1,21]. Although we had planned to compare results from different ZC crown brands, the sample size was too small. We might infer from the positive parental evaluation of all ZC brands in the study and their high success rate that there might be no significant difference among brands but a larger study group in a future study would be required to confirm that. Other limitations of the study were that the dentist evaluators made their rating based upon clinical photographs, while parents looked directly at their children. While many photographic images can be excellent, differences in lighting and contrast can provide an image that may be more or less aesthetic than the clinical evaluation. Although variables in lighting and digital processing can impact the shade of the crowns for evaluation, the impact should be similar on the crowns and adjacent unrestored teeth but might impact the examiner evaluations from subject to subject. There should be no impact of the photographic evaluation on crown contour. As a flash was not used ambient lighting conditions may have led to inconsistent colour in the captured images. A photographic system that controlled all variables for a consistent result will be suggested in the future. Additionally, the dentist evaluators examined photos that were close-ups of the restored teeth, while parents were likely to evaluate the teeth from a distance of 12–18 inches. At the follow-up evaluation when the questionnaires were given to the parents, the operator dentist who had placed the crowns was also present to answer questions regarding the parental evaluation. It is possible that his presence may have caused parents to be less judgmental, perhaps not wanting to criticize the dentist’s work in front of him. 

This study was able to evaluate teeth at an average time of 13.3 months, slightly longer than two other studies and with a similar number of teeth evaluated, with parent satisfaction in all studies consistently high [15,16]. The number of teeth and subjects was much greater but the duration of placement less than in another study, but again parent satisfaction was quite high in both studies [9]. Both prospective and retrospective studies had similar results and all used parental questionnaires completed with the patient present and the assistance of a dentist in the clinic to answer any questions, which may have affected parent responses in all studies. This study utilized only one dental provider, which may have affected results compared to multiple providers in other studies, although they were calibrated.

## 5. Conclusions

Based upon the results of this study the following conclusions can be made. Overall parental satisfaction with zirconia crowns for the restoration of maxillary anterior primary incisors was excellent and no statistical difference was noted among crown brands. Parents would choose zirconia crowns again and also recommend them to a friend. Parents were less critical of colour match and crown contour than dentist evaluators and indicated a high level of satisfaction.

## Figures and Tables

**Table 1:** Assessment criteria for parents and dental evaluators.

Assessment Criteria	Parent	Dental Evaluators
**Colour Match**		
1	There is no difference from other teeth	No noticeable difference from adjacent teeth
2	There is a slight mismatch from adjacent teeth	Slight shade mismatch
3	There is an obvious mismatch from adjacent teeth	Obvious shade mismatch
**Crown Contour**		
1	Crown nicely contoured and natural-looking	Crown appears very cosmetic, nicely contoured and natural-looking
2	Crown acceptable but could have better shape	Crown appears acceptable but could have been contoured better, perhaps longer, shorter, wider, thinner
3	Crown unacceptable	Crown not aesthetic, detracts from the appearance of the mouth
**Crown Durability**		
1	Crown appears intact with no chips, cracks or fracture	Crown appears normal; no cracks, chips or fracture
2	Crown has small, noticeable chips, cracks or fracture	Small but noticeable areas of loss of material
3	Crown has large chips, cracks, fracture	Large loss of crown material
4	Crown is missing 1 lost naturally 2 trauma 3 extraction due to infection	
5	Crown has been repaired or replaced	
**Overall Parent** **Satisfaction**		
Yes or No	Overall, were you satisfied with the results of the crowns	
Yes or No	Would you choose this procedure if once again offered	
Yes or No	Would you recommend this procedure to a friend with a child having a similar problem	

**Table 2:** Demographic data of study subjects.

Patients	37 (Male = 27, Female = 10)
Teeth	131
Cheng Crowns	22 (7 cases)
NuSmile Crowns	91 (25 cases)
Sprig Crowns	18 (5 cases)
Pulpotomy	15
Age at treatment	41.5 months
Recall duration mean time	13.3 months
Short term recall group duration	6–10.4 months (mean 8.1, n = 22)
Long term recall group duration	12.5–33.8 months (mean 21.0, n = 15)
Crown retention rate	99.7% (1 crown rebonded at 2 months)
Sedation	16 patients, 53 crowns
General anaesthesia	21 patients, 78 crowns

**Table 3:** Colour match rank by parents and dental evaluators.

Colour Match	Rank 1	Rank 2 or 3 *
Parent	84% (n = 95)	26% (n = 36)
Dental Evaluators	36% (n = 47)	64% (n = 84)

* Only 5 teeth were ranked 3 and only by dental evaluators. No significant difference in colour match between parent and dentist (Chi-square *p* < 0.05).

**Table 4:** Crown contour rank by parents and dental evaluators.

Crown Contour	Rank 1	Rank 2 or 3 *
Parent	97% (*n* = 142)	3% (*n* = 4)
Dental Evaluators	55% (*n* = 73)	45% (*n* = 58)

* Only 12 teeth were ranked 3 and only by dental evaluators; No significant difference in crown contour ranking between parent and dentist (Chi-square, *p* < 0.05).

**Table 5:** Colour match ranking by parent or dental evaluator for short-term and long-term follow-up.

Colour Match Parent vs. Dentist	Short Term Recall Group Recall	Long Term Recall Group Recall
*n* = 131 crowns	Mean recall 8.1 month	Mean recall 21.0 months
Parent		
Rank 1	61	34
Rank 2	20	16
Dental Evaluators		
Rank 1	29	18
Rank 2 or 3	52	32

No significant difference in rank due to mean length of follow-up in either group (Chi-square, *p* < 0.05).

**Table 6:** Colour match ranking by parent or dental evaluator for pulpotomized teeth.

Colour Match Pulpotomized Teeth	Pulpotomy (*n* = 15)	No Pulpotomy (*n* = 13)
Parent		
Rank 1	35.7% (*n* = 10)	35.7% (*n* = 10)
Rank 2	17.9% (*n* = 5)	10.7% (*n* = 3)
Dental Evaluator		
Rank 1	7.1% (*n* = 2)	17.9% (*n* = 5)
Rank 2 or 3	46.4% (*n* = 13)	28.6% (*n* = 8)

Only two teeth were ranked 3 in only one subject by dental evaluators; No statistically significant difference in colour match ranking due to pulp therapy in either group (Chi-square, *p* < 0.05).

## Data Availability

The data supporting the reported results resides with the corresponding author.
